# Supplementary material for: Characterization of a Temporal Profile of Biomarkers as an Index for Ischemic Stroke Onset Definition
Source: J Clin Med. 2021 Jul 15;10(14):3136. doi: 10.3390/jcm10143136 (PMC8307571; doi:10.3390/jcm10143136)
Supplement: Supplementary file 1 [file jcm-10-03136-s001.zip › jcm-1247148-supplementary.pdf]

**Table S1.** Univariate analysis of demographics aspects using latency times as dependent variable.

| | Q1 | Q2 | Q3 | Q4 | *p* |
|---|---|---|---|---|---|
| Age, years | 70.4 ± 13.5 | 72.4 ± 14.2 | 72.2 ± 13.7 | 72.4 ± 13.3 | 0.026 |
| Previous Rankin scale | 0 (0, 1) | 0 (0, 1) | 0 (0, 0) | 0 (0, 1) | 0.338 |
| Female sex, % | 24.1 | 56.1 | 51.2 | 49.6 | <0.0001 |
| Arterial hypertension, % | 59.3 | 67.1 | 63.7 | 60.8 | 0.003 |
| Diabetes, % | 22.3 | 23.0 | 25.3 | 26.3 | 0.160 |
| Smoking, % | 20.3 | 13.9 | 14.2 | 16.7 | 0.001 |
| Embolism, % | 15.5 | 9.0 | 10.4 | 11.1 | <0.0001 |
| Hyperlipidaemia, % | 35.3 | 36.7 | 33.6 | 33.4 | 0.428 |
| Peripheral arteria disease, % | 7.1 | 5.2 | 5.4 | 5.8 | 0.305 |
| Ischemic heart disease, % | 11.4 | 10.4 | 13.3 | 11.1 | 0.250 |
| Heart failure, % | 4.8 | 4.3 | 3.8 | 4.2 | 0.799 |
| Atrial fibrillation, % | 20.5 | 21.7 | 21.3 | 20.8 | 0.925 |
| Carotid disease, % | 1.8 | 1.4 | 1.5 | 1.5 | 0.940 |
| Carotid recanalization, % | 0.4 | 0.3 | 0 | 0.2 | 0.271 |
| Transient ischemic attack, % | 4.8 | 6.1 | 5.5 | 4.3 | 0.332 |
| TIA time | | | | | 0.234 |
| <1 day, % | 2.9 | 4.7 | 3.7 | 3.1 | |
| 1–7 days, % | 1.3 | 1.0 | 0.7 | 0.7 | |
| >7 days, % | 0.3 | 0.3 | 0.9 | 0.5 | |
| Previous stroke, % | | | | | 0.324 |
| Ischemic stroke, % | 14.6 | 14.0 | 13.2 | 15.1 | |
| Intracerebral haemorrhage, % | 0.4 | 1.0 | 1.5 | 1.3 | |
| Antiplatelets, % | 22.7 | 25.8 | 25.8 | 24.6 | 0.391 |
| Anticoagulant, % | 9.1 | 8.4 | 6.6 | 7.8 | 0.238 |

**Table S2.** Glutamate and interleukin-6 univariate analysis in stroke patients subdivided by different latency times.

| | Q1 | Q2 | Q3 | Q4 | p |
|---|---|---|---|---|---|
| Glutamate, µM/mL | 297.9 ± 140.1 | 120.9 ± 113.7 | 61.3 ± 82.3 | 138.8 ± 119.7 | <0.0001 |
| IL6, pg/mL | 12.8 ± 16.5 | 32.8 ± 14.6 | 30.7 ± 14.7 | 34.4 ± 14.9 | <0.0001 |

**Table S3.** Glutamate and interleukin-6 univariate analysis in stroke patients (not including awakening strokes) subdivided by different latency times.

| | Q1 | Q2 | Q3 | Q4 | p |
|---|---|---|---|---|---|
| Glutamate, µM/mL | 302.7 ± 88.6 | 128.2 ± 53.9 | 60.6 ± 64.8 | 134.5 ± 90.2 | <0.0001 |
| IL6, pg/mL | 18.1 ± 12.4 | 29.6 ± 13.3 | 31.8 ± 13.5 | 35.4 ± 11.4 | <0.0001 |

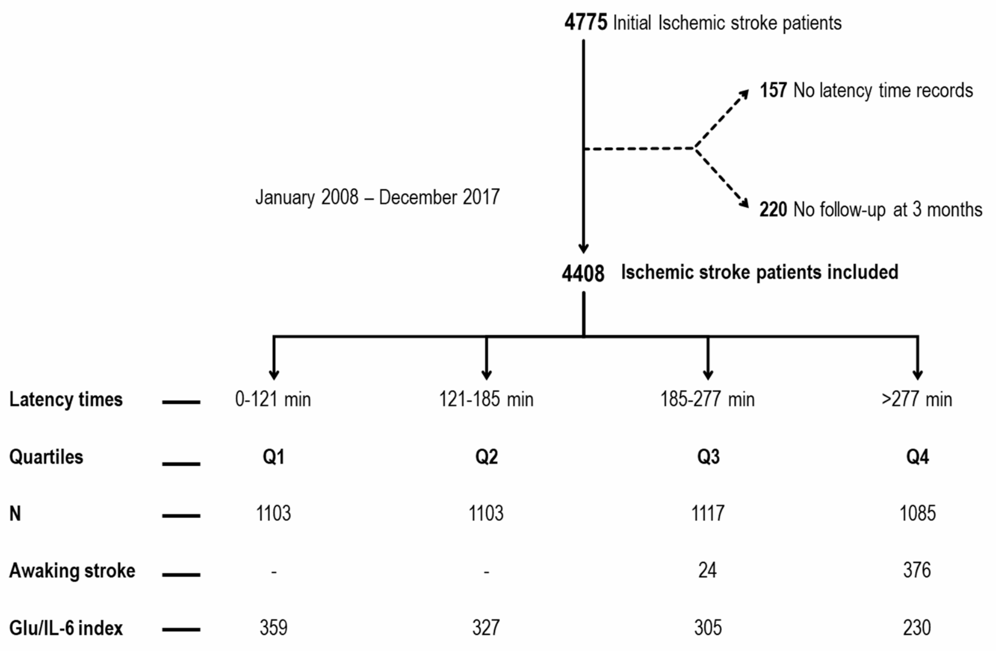


**Figure S1.** Study design flowchart.
